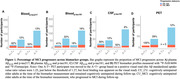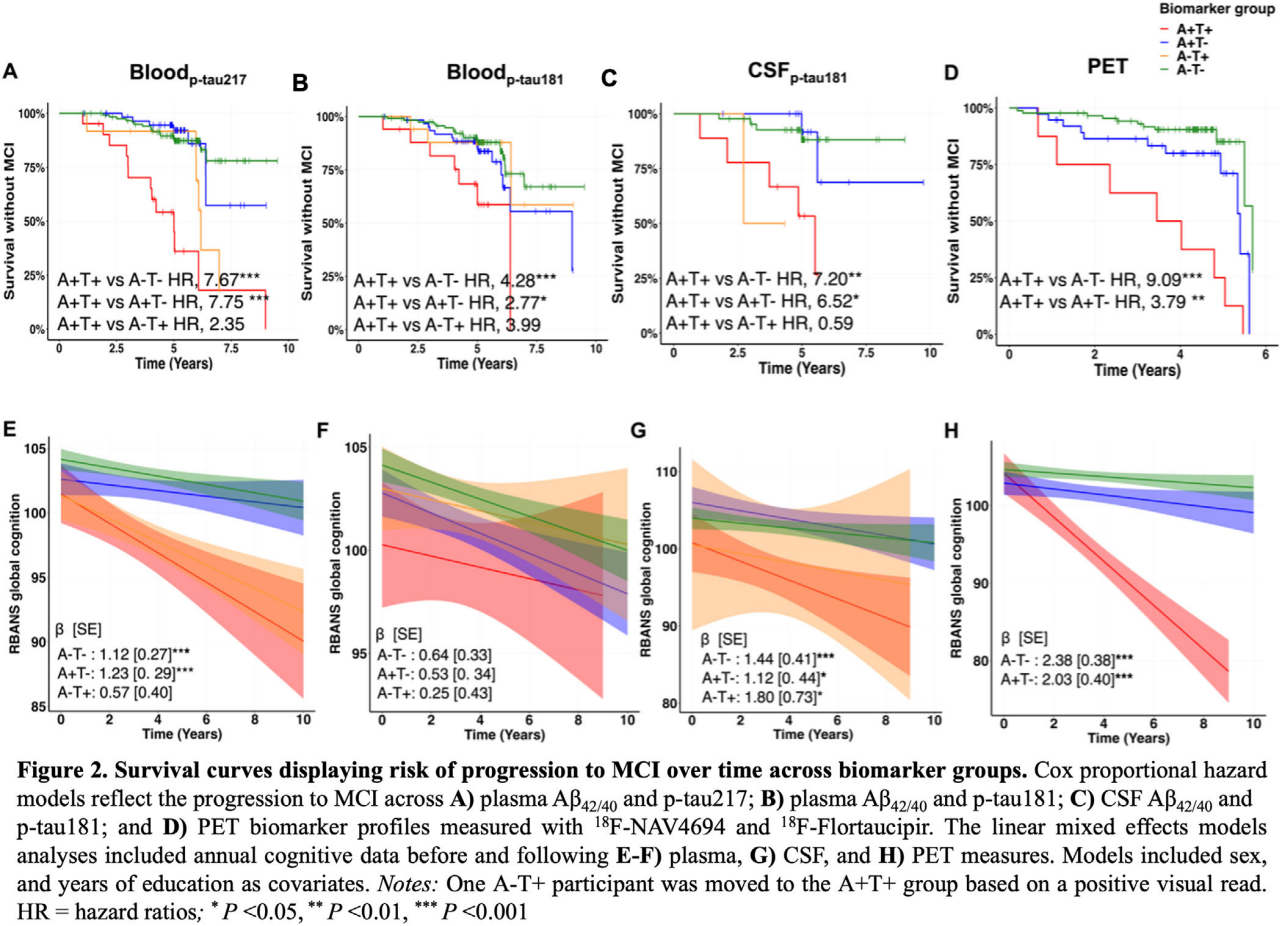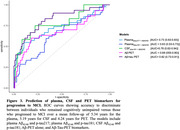# The role of biofluid markers in predicting near‐term cognitive impairment

**DOI:** 10.1002/alz.094038

**Published:** 2025-01-09

**Authors:** Yara Yakoub, Fernando Gonzalez‐Ortiz, Nicholas J. Ashton, Thomas K Karikari, Cherie Strikwerda‐Brown, Frédéric St‐Onge, Valentin Ourry, Michael Schöll, Maiya R. Geddes, Simon Ducharme, Pedro Rosa‐Neto, Jean‐Paul Soucy, John C.S. Breitner, Henrik Zetterberg, Kaj Blennow, Judes Poirier, Sylvia Villeneuve

**Affiliations:** ^1^ Douglas Mental Health University Institute, Centre for Studies on the Prevention of Alzheimer's Disease (StoP‐AD), Montréal, QC Canada; ^2^ Institute of Neuroscience and Physiology, University of Gothenburg, Mölndal Sweden; ^3^ Centre for Age‐Related Medicine, Stavanger University Hospital, Stavanger Norway; ^4^ Department of Psychiatry, School of Medicine, University of Pittsburgh, Pittsburgh, PA USA; ^5^ Department of Psychiatry and Neurochemistry, Institute of Neuroscience and Physiology, The Sahlgrenska Academy, University of Gothenburg, Mölndal Sweden; ^6^ Montreal Neurological Institute, McGill University, Montréal, QC Canada; ^7^ Montreal Neurological Institute, McGill University, Montreal, QC Canada; ^8^ McGill Center for Research Studies in Aging, Montréal, QC Canada; ^9^ Department of Psychiatry and Neurochemistry, Institute of Neuroscience and Physiology, The Sahlgrenska Academy, University of Gothenburg, Mölndal, Gothenburg Sweden

## Abstract

**Background:**

PET biomarkers have proven valuable for identifying cognitively unimpaired (CU) individuals at‐risk of near‐term clinical progression. Given the increasing interest in plasma biomarkers to detect Alzheimer’s pathology, we assessed levels of amyloid (Aß42/40) and tau (p‐tau217 and p‐tau181) biomarkers in plasma (A+T+plasma) in CU individuals as predictors of clinical progression to mild cognitive impairment (MCI). We then repeated these analyses using cerebrospinal fluid (CSF) and PET biomarkers.

**Method:**

We studied 218 participants from the PREVENT‐AD cohort with cognitive follow‐up (mean 5.34 years, range 1–10 years) and plasma biomarkers available while CU. Cognition was assessed annually using the RBANS. Plasma biomarkers were measured using IP‐MS for Aß42/40 and in‐house single molecular arrays for p‐tau217 and p‐tau181. An overlapping 71 participants had Aß42/40 (Mesoscale Discovery assay) and p‐tau181 (Innotest immunoassay) CSF data available, and 135 participants had Aß (18F‐NAV4694) and tau (18F‐florbetapir) PET data. We also performed receiver operating characteristic (ROC) curves to compare the performance of plasma (Aß42/40 and p‐tau217; Aß42/40 and p‐tau181), CSF (Aß42/40 and p‐tau181) or PET (Aß‐ and tau‐PET) biomarkers in predicting progression to MCI. We examined an additional model including only Aß‐PET, given the importance of this technique as a stand‐alone marker for recruitment in anti‐Aß trials.

**Result:**

The proportion who progressed from CU to MCI was 62% in individuals with A+T+plasma217; and 41% with A+T+plasma181; 56% with A+T+CSF181 and 100% with A+T+PET (Figure 1). Cox proportional hazard models indicated a faster progression rate in all A+T+ groups compared with their matched A‐T‐ biomarker groups (Figure 2). Considering longitudinal RBANS scores, all A+T+ groups declined faster than the other groups, except for the plasma181 group. Finally, the PET (Aß and tau‐PET) and CSF models were superior to the plasma or Aß‐PET alone in identifying progressors, with no meaningful difference between the Aß‐PET and the biofluid models (Figure 3).

**Conclusion:**

CU individuals with A+T+ based on plasma biomarkers are at increased risk of cognitive decline and clinical progression. Nevertheless, the proportion who progressed to MCI in individuals classified with plasma was lower than that found using PET. Thus, the latter should remain the gold standard to identify presymptomatic pathological changes.